# Aberrant MUC1-TRIM46-KRTCAP2 Chimeric RNAs in High-Grade Serous Ovarian Carcinoma

**DOI:** 10.3390/cancers7040878

**Published:** 2015-10-19

**Authors:** Kalpana Kannan, Gona Karimi Kordestani, Anika Galagoda, Cristian Coarfa, Laising Yen

**Affiliations:** 1 Department of Pathology & Immunology, Baylor College of Medicine, Houston, TX 77030, USA; kalpana.kann@gmail.com (K.K.); Gonakarimi188@gmail.com (G.K.K.); anikagalagoda@gmail.com (A.G.); 2 Department of Molecular and Cellular Biology, Baylor College of Medicine, Houston, TX 77030, USA; coarfa@bcm.edu; 3 Dan L. Duncan Cancer Center, Baylor College of Medicine, Houston, TX 77030, USA; 4 Center for Drug Discovery, Baylor College of Medicine, Houston, TX 77030, USA

**Keywords:** chimeric RNA, high-grade serous ovarian cancer, MUC1, TCGA, RNA-seq

## Abstract

High-grade serous ovarian cancer (HGSC) is among the most lethal forms of cancer in women. By analyzing the mRNA-seq reads from The Cancer Genome Atlas (TCGA), we uncovered a novel cancer-enriched chimeric RNA as the result of splicing between MUC1, a highly glycosylated transmembrane mucin, TRIM46, a tripartite motif containing protein, and KRTCAP2, a keratinocyte associated protein. Experimental analyses by RT-PCR (reverse transcription PCR) and Sanger sequencing using an in-house cohort of 59 HGSC patient tumors revealed a total of six MUC1-TRIM46-KRTCAP2 isoforms joined by different annotated splice sites between these genes. These chimeric isoforms are not detected in non-cancerous ovaries, yet are present in three out of every four HGSC patient tumors, a significant frequency given the exceedingly heterogeneous nature of this disease. Transfection of the cDNA of MUC1-TRIM46-KRTCAP2 isoforms in mammalian cells led to the translation of mutant MUC1 fusion proteins that are unglycosylated and cytoplasmically localized as opposed to the cell membrane, a feature resembling the tumor-associated MUC1. Because the parental MUC1 is overexpressed in 90% of HGSC tumors and has been proposed as a clinical biomarker and therapeutic target, the chimeric MUC1-TRIM46-KRTCAP2 isoforms identified in this report could represent significantly better MUC1 variants for the same clinical utilities.

## 1. Introduction

High-grade serous ovarian cancer (HGSC) is the most lethal gynecological malignancy and the fifth most common cause of cancer-related deaths in women in the USA [1]. The high mortality rate of this cancer is due to the fact that most patients are diagnosed at advanced stages. Novel molecular signatures that enable better detection for this deadly disease while it is still surgically treatable will greatly increase survival rate.

Chimeric RNAs are aberrant RNA transcripts possessing sequences from different genes. They are generated by either “read-through/splicing” or “trans-splicing” [2]. Although the exact cause and regulation of chimeric RNAs are unknown, chimeric RNAs are expected to increase the proteomic diversity in cells through chimeric proteins or altered regulation of participating mRNAs [3,4,5]. Recent studies suggest that chimeric RNAs are more common than previously thought, and more abundant in cancer than in non-cancer cells [6]. This raises the possibility that increased chimeric RNA events could represent a new class of molecular alteration in cancer. Despite the potential importance, little is known about chimeric RNAs in HGSC. In this report, we took advantage of The Cancer Genome Atlas (TCGA) transcriptome sequencing data to identify MUC1-TRIM46-KRTCAP2 as a novel and cancer-enriched chimeric RNA in HGSC.

MUC1 (Mucin1, also known as CA15-3) is a member of the highly glycosylated transmembrane mucin family. MUC1 is a heterodimer of two subunits that are non-covalently held together. The larger extracellular subunit is characterized by a signal peptide domain and a polymorphic domain called variable number of tandem repeats (VNTR) [7]. This VNTR domain contains several serine and threonine residues that are heavily glycosylated, giving MUC1 a large molecular mass. The smaller subunit has a short extracellular domain, a SEA domain, a single-pass transmembrane domain and a cytoplasmic tail [7]. After translation, MUC1 protein is cleaved at the SEA domain into two subunits in the endoplasmic reticulum. The cytoplasmic tail contains several phosphorylation sites that are important for its role in intracellular signaling as well as a motif for homodimerization of the smaller subunit [7]. While various MUC1 splicing isoforms have been reported [8], no MUC1 chimeric RNA has been found to date.

MUC1 is typically found on the apical surface of epithelial cells lining various tissues, and is thought to provide hydration, lubrication, protection from proteases and defense against pathogens to the underlying epithelia. It is overexpressed in about 90% of HGSC tumors patients [9], possibly disrupting cell–cell and cell–matrix adhesions [10,11], thereby facilitating invasive growth and metastasis. The high levels of MUC1 are associated with an advanced cancer grade/stage of ovarian carcinoma [12]. In tumor cells, MUC1 is underglycosylated and located in the cytoplasm in addition to the cell membrane, and this depolarized expression is associated with poor prognosis [13]. A recent study using a triple transgenic MUC1/Kras/Pten mouse model also showed that MUC1 positive ovarian tumors have a greater capacity for metastatic spread [14]. Furthermore, the overproduction of MUC1 in ovarian cancer leads to the increased circulating serum MUC1 levels detectable by standardized ELISA tests [15]. These features make MUC1 an attractive biomarker as well as a possible therapeutic target. However, the considerable variability in MUC1 expression among tumor and normal samples [16] has made it difficult to be used as a reliable clinical biomarker.

In this report, we utilized the TCGA high-throughput transcriptome sequencing data to identify MUC1-TRIM46-KRTCAP2 as a novel, cancer-enriched chimeric RNA in HGSC. This aberrant RNA has six splicing isoforms and they are expressed in three of every four HGSC tumors. We demonstrated that ectopic expression of these chimeric RNAs results in fusion proteins with a novel amino acid sequence. Moreover, MUC1-TRIM46-KRTCAP2 fusion isoforms are unglycosylated and localized in the cytoplasm as opposed to the cell membrane, a feature resembling tumor-associated MUC1. Unlike parental MUC1 that is present in both normal ovary and cancer cells, MUC1-TRIM46-KRTCAP2 chimeric RNAs are detected primarily in tumors, and thus may represent significantly better MUC1 variants for cancer detection using local biomaterials such as biopsy tissues.

## 2. Results

### 2.1. MUC1-TRIM46-KRTCAP2 Is a Recurrent Chimeric RNA in HGSC

The Cancer Genome Atlas (TCGA) has performed extensive high-throughput transcriptome sequencing on HGSC patient samples [17]. For our analysis, we utilized the sequencing reads from 130 HGSC patient samples, a sample size that is sufficient to identify highly recurrent chimeric RNAs. Our strategy for identifying chimeric transcripts was to search for paired “chimeric” reads with each read mapping to a different gene either in the genome or transcriptome using our previously established pipeline [18]. This strategy led to the identification of nearly 1383 chimeric RNA candidates from the 130 cancer samples. We selected 33 out of 1383 chimeric RNA candidates for experimental validation based on the criteria that the chimeric RNA must be present in at least five cancer samples. To experimentally validate these candidates, we designed specific primers with each primer targeting one parental gene, therefore specifically amplifying only the chimeric RNA but not the parental gene transcripts. For 9 of the 33 chimeric RNA candidates, we were able to obtain RT-PCR (reverse transcription PCR) products from in-house tumor samples. The RT-PCR bands were excised and sequenced by Sanger sequencing. This led to the confirmation of the presence of nine chimeric RNAs, and the identification of their exact RNA junction (Table S1). Since TCGA transcriptome sequencing collection does not contain organ specific controls, we examined these nine chimeric RNAs using our in-house non-cancerous ovary samples to answer the question of whether these candidates are also present in non-cancerous tissue in addition to the cancer samples. The frequency of occurrence for the nine chimeric RNAs in non-cancerous ovary samples is listed in Table S2. Among them, MUC1-TRIM46 is the only chimeric RNA that was highly recurrent in HGSC tumor samples but absent in non-cancer samples (see below).

**Figure 1:**
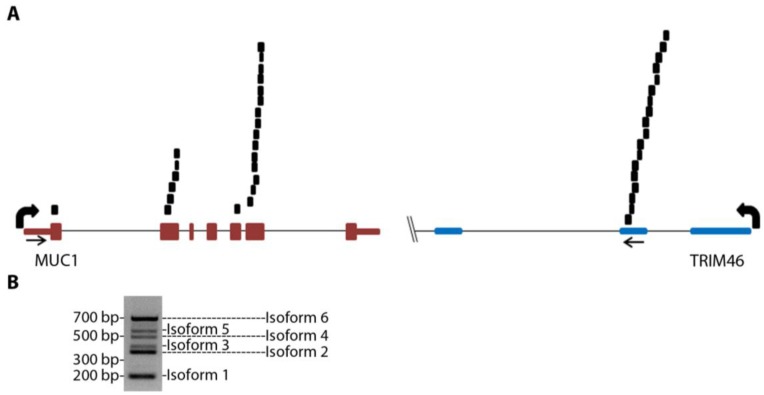
MUC1 chimeric RNAs identified in TCGA database of HGSC patient samples. (**A**) Schematic showing the position of 25 paired chimeric reads aligning to both MUC1 and TRIM46 genes identified from nine patients in the 130 TCGA cohort. Arrows indicate PCR primer targeting locations. (**B**) An example of RT-PCR validation for MUC1-TRIM46 chimeric RNA using one of the in-house HGSC patient samples. The bands corresponding to the six isoforms are labeled as shown.

**Figure 2:**
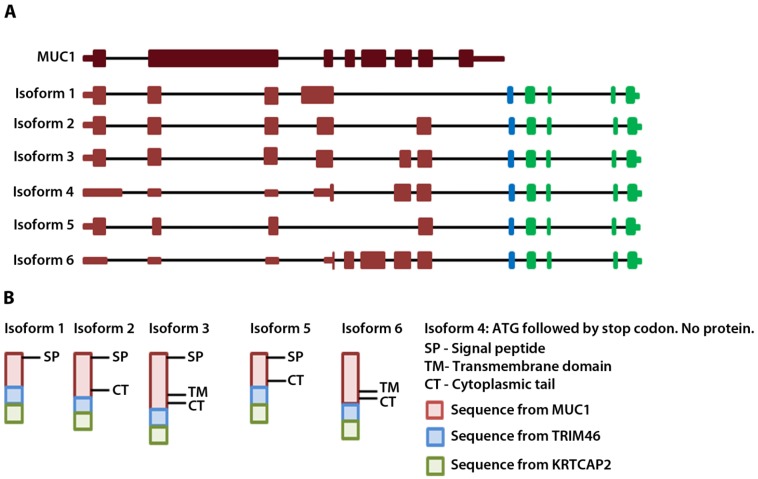
MUC1-TRIM46-KRTCAP2 chimeric RNA isoforms and predicted protein consequences. (**A**) Schematic of the parental MUC1 and the six isoforms of MUC1-TRIM46-KRTCAP2 chimeric RNAs are shown with MUC1 (red), TRIM46 (blue) and KRTCAP2 (green) regions. The indicated coding and non-coding regions of MUC1 are based on the annotation of specific transcripts in the UCSC genome browser. (**B**) The expected protein products of these chimeric RNAs are shown with the domains indicated.

MUC1-TRIM46 chimeric RNA is novel and has not been previously reported. The presence of this fusion transcript in TCGA cancer samples is supported by 25 paired “chimeric” reads with one read mapping to MUC1 and the other to TRIM46 (Figure 1A and Table S3, using GRCh37/hg19). RT-PCR analysis using several in-house cancer samples (see Table S4 for primers) resulted in six bands of different sizes (an example is shown in Figure 1B). These bands were gel purified, cloned, subjected to Sanger sequencing, and confirmed to harbor different exons of MUC1 joined to the same genomic sequence of TRIM46 by annotated splice sites (Figure 2A). This indicates that the RNA junctions of these chimeric RNAs are the result of splicing.

To annotate the ends of the MUC1-TRIM46 chimeric RNAs, we analyzed available EST collections. The analysis revealed two ESTs (CD366871.1 and BF870262.1) that contain a sequence from either MUC1 or TRIM46 extending into a third gene KRTCAP2. To test if the chimeric RNAs we identified indeed extend into KRTCAP2, we designed a primer pair to amplify from the beginning of the 5’ UTR of MUC1 to the very end of the 3’ UTR of KRTCAP2. RT-PCR performed on cancer samples yielded six full-length bands. Sequencing of cloned bands revealed that they correspond to the six isoforms described in Figure 1B, but now with confirmed mRNA sequences extending from the 5’ end of MUC1 till the 3’ end of KRTCAP2 with one exon in between originating from the TRIM46 sequence (Figure 2A, Table S5-sequences). The six isoforms differ in the MUC1 region where different exons are recruited but they all have an identical TRIM46 and KRTCAP2 sequence (Figure 2A). Two unique RNA fusion junctions were identified among the six isoforms (Table S5). One is unique to isoform 1 and the other is common among isoforms 2–6. Henceforth, these chimeric RNAs are referred to as MUC1-TRIM46-KRTCAP2 instead of MUC1-TRIM46.

To test whether the transcription of MUC1-TRIM46-KRTCAP2 chimeric RNA is the result of a genomic segment deletion between MUC1 and TRIM46 gene, we employed the same primer pair used in Figure 1A to detect the presence of genomic deletion from HGSC patients’ genomic DNA. However, genomic DNA PCR showed the same size bands as compared to reference human genomic DNA (data not shown), suggesting that the chimeric RNAs are not the result of genomic rearrangement, but are generated at the transcriptional level either by trans-splicing or read-through splicing.

**Figure 3:**
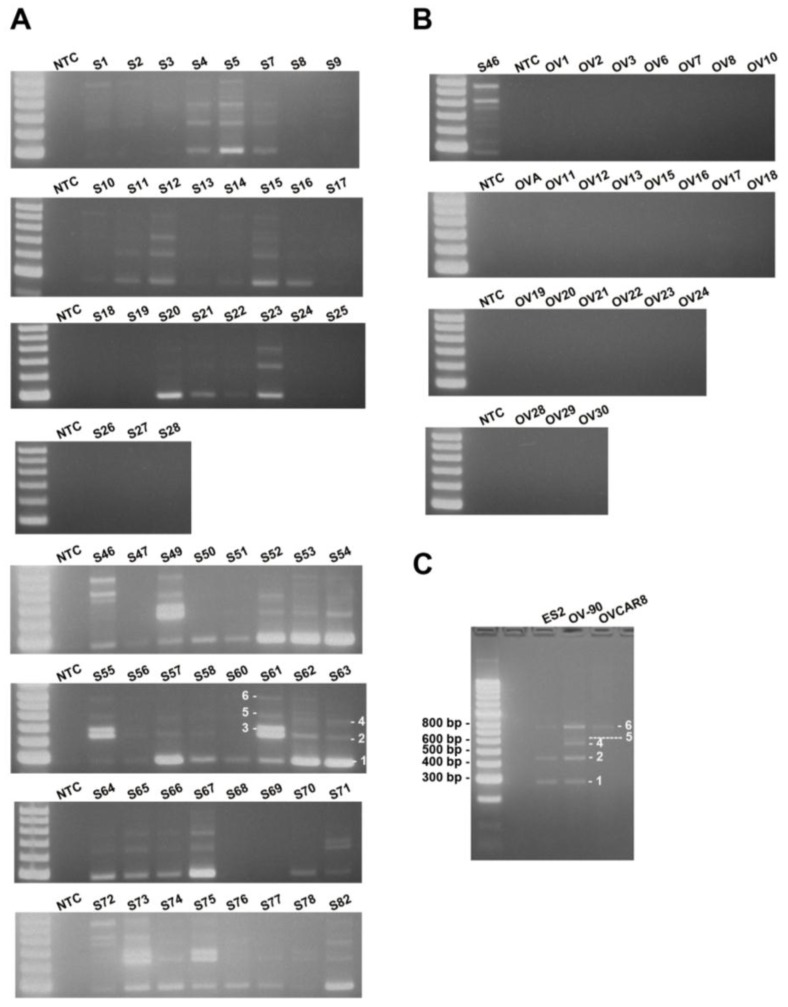
MUC1-TRIM46-KRTCAP2 is a highly recurrent chimeric RNA in HGSC patient tumor samples and cell lines. (**A**) The results of RT-PCR for MUC1-TRIM46-KRTCAP2 in 59 HGSC tumor samples (denoted by “S”). (**B**) The results of 24 non-cancerous ovary samples (“OV”) are shown. NTC refers to “no cDNA control”. The different isoforms are indicated on samples S61 and S63. (**C**) The results of RT-PCR for MUC1-TRIM46-KRTCAP2 in three HGSC cell lines (ES2, OV90 and OVCAR8) are shown.

To estimate the frequency of occurrence of MUC1-TRIM46-KRTCAP2 chimeric RNAs, we performed RT-PCR on a cohort of 59 HGSC patient samples. The results showed that MUC1-TRIM46-KRTCAP2 chimeric RNAs are highly recurrent with 75% of the cancer samples containing at least one isoform (Figure 3A). MUC1-TRIM46-KRTCAP2 isoform 1 is the most common chimeric RNA and is observed in 64% of the cancer samples, while isoform 3 is the least common chimeric RNA and is expressed in 17% of the cohort (Table S6). In contrast, none of the isoforms are detected in the 24 non-cancerous ovary samples (Figure 3B). Thus, the results suggest that MUC1-TRIM46-KRTCAP2 chimeric RNAs are highly cancer-enriched.

Since MUC1-TRIM46-KRTCAP2 family has a high frequency of occurrence among cancer samples, we speculated that this chimeric RNA might also be present in established HGSC cell lines. This indeed is the case. By RT-PCR screening of three serous type cancer cell lines (ES2, OV-90 and OVCAR8), we found that isoforms of MUC1-TRIM46-KRTCAP2 are expressed in all three cell lines (Figure 3C). However, different isoforms are selectively expressed in each cell line, a pattern also found in patient samples. The presence of these chimeric RNAs in established serous type cancer cell lines further support the potential significance of the MUC1-TRIM46-KRTCAP2 in HGSC.

### 2.2. MUC1-TRIM46-KRTCAP2 Isoforms Are Translated into Intracellular Fusion Proteins

The complete cDNA sequence of MUC1-TRIM46-KRTCAP2 obtained from RT-PCR enabled us to predict the protein consequences of these highly recurrent chimeric RNAs. Isoform 4 is predicted to yield no protein since the annotated start codon is immediately followed by a stop codon. The remaining five isoforms are predicted to translate different fusion proteins with the MUC1 C-terminal domain replaced by a common sequence composed of 53 amino acids from TRIM46 and 54 amino acids from KRTCAP2 (Figure 2B and Table S7). However, the TRIM46 sequence in the chimeric RNAs is in the antisense direction and the KRTCAP2 sequence is out of frame. Therefore, these newly added common amino acids are unrelated to parental TRIM46 or KRTCAP2 proteins. These predicted protein isoforms lack the VNTR region, which is the site of glycosylation in parental MUC1 (Figure 2B). The signal peptide is present in isoforms 1, 2, 3 and 5, while the transmembrane domain is present only in isoforms 3 and 6. The MUC1 cytoplasmic tail is maintained in all isoforms except for isoform 1. The differences in the domains retained in the isoforms suggest that they could result in varied cellular localization.

**Figure 4:**
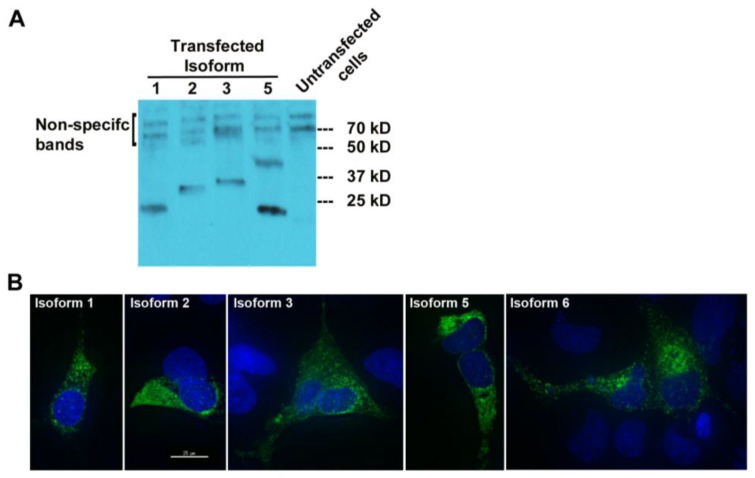
MUC1-TRIM46-KRTCAP2 chimeric RNAs give rise to fusion proteins. (**A**) MUC1-TRIM46-KRTCAP2 isoforms were cloned with a C-terminal FLAG tag and expressed in OVCAR8 cells. Western blot of protein extracts using FLAG antibody shows that most of the isoforms are translated with the expected sizes lacking glycosylation. Isoform 5 appears to form a homodimer. (**B**) Immunocytochemistry of OVCAR8 cells transfected with different MUC1-TRIM46-KRTCAP2-FLAG expression constructs. The fusion protein isoforms are seen mainly in the cytoplasm as visualized by FLAG antibody. Images were taken using deconvolution microscopy.

To check whether MUC1-TRIM46-KRTCAP2 chimeric RNAs are translated into fusion proteins, we tagged the full-length cDNA of five isoforms (1, 2, 3, 5 and 6) with a FLAG at the C-terminus. The predicted sizes for the isoforms are between 23 and 30 kD (Figure 2A). Transfection in OVCAR8 cell line and the subsequent Western blot analysis revealed that all isoforms are translated with the exception of isoform 6 (Figure 4A). The results confirmed that most of the MUC1-TRIM46-KRTCAP2 fusion proteins are translated as predicted. However, we are not able to confirm the presence of endogenous MUC1-TRIM46-KRTCAP2 isoforms expressed in tumor tissue and in cancer cell lines because (1) most of the commercially available antibodies target the VNTR domain that is lacking in our fusion protein isoforms and (2) the sizes of these fusion protein isoforms are very similar to the various protein isoforms of parental MUC1, making it difficult to conclusively distinguish between the two groups.

To test whether these translated protein isoforms are secreted in the media, we performed Western blotting analyses using the culture media collected from transfected cells. As shown in Figure S1, none of the MUC1-TRIM46-KRTCAP2 isoforms were detected in the culture media, indicating they are not secreted. Immunocytochemical imaging using anti-FLAG antibody showed that the MUC1-TRIM46-KRTCAP2 fusion proteins are mainly located in the cytoplasm as opposed to the cell membrane of transfected OVCAR8 cells (Figure 4B). Surprisingly, isoform 6, which is not detected by Western blotting, is present in the cytoplasm by immunocytochemistry. This suggests that isoform 6 is also translated but the FLAG epitope can only be detected when the protein is in the native conformation. Alternatively, the FLAG epitope may be cleaved off from isoform 6, resulting in a fragment too small to be detected on the Western blot. The cytoplasmic localization of these isoforms is in contrast to parental MUC1, which is a transmembrane protein, indicating that MUC1-TRIM46-KRTCAP2 fusion proteins may have protein functions that are very different from parental MUC1 protein functions.

## 3. Discussion

High-grade serous ovarian cancer is among the most lethal forms of cancer in women. MUC1 is overexpressed in about 90% of HGSC patients making it an attractive biomarker as well as a therapeutic target. However, the considerable variability in the expression of MUC1 among normal and tumor samples as evidenced by different detection methods [16] has made it challenging to use in clinical settings. By utilizing the The Cancer Genome Atlas (TCGA) transcriptome database of HGSC patient samples [17], we identified a family of aberrant MUC1-associated chimeric RNAs that are highly enriched in HGSC. Several key features are associated with this family of chimeric RNAs. First, the chimeric RNAs are present in every three out of four HGSC tumors (75% occurrence rate), a significant frequency given the exceedingly heterogeneous nature of this malignancy. Second, it is highly cancer-enriched and is not detectable in non-cancerous ovaries. Third, the chimeric RNA family has at least six splicing isoforms, all missing the 3’ UTR of parental MUC1. Fourth, the new 3’ RNA sequence derived from TRIM46 and KRTCAP2 gives rise to a new C-terminus amino acid sequence that is identical among the isoforms, suggesting a possible gain of function. Lastly, unlike the parental MUC1 proteins, these protein isoforms are localized in the cytoplasm. Together, these features suggest that MUC1-TRIM46-KRTCAP2 may have important biological roles and potential clinical utilities in HGSC.

The VNTR domain is known to contain many serine and threonine residues that are heavily glycosylated, giving MUC1 a large molecular mass. None of the fusion isoforms contain this VNTR domain, suggesting that these protein isoforms are not glycosylated thus having much smaller molecular weight. This is confirmed by Western blotting, wherein we did not observe a large molecular weight band for these isoforms. The secreted form of parental MUC1 is a short protein containing only the VNTR domain and the signal peptide [7]. Although isoforms 1, 2, 3 and 5 retain the signal peptide, they all lack the VNTR region, and our results show that they are not secreted into the media. The GSVVV motif located within the SEA domain, where parental MUC1 is cleaved, is present only in isoform 6. However, this isoform is undetectable by Western blot analysis and hence it is unknown whether it gets cleaved like parental MUC1. The motif for homo-dimerization is located in the cytoplasmic tail [7], which is present in isoform 2, 3, 5 and 6. However, only isoform 5 appears to form a homodimer (Figure 4A) and the reason for this is unclear. It is also possible that this band is the result of post translational modifications, but not dimerization. In addition, the presence (isoform 3 and 6) or absence (isoform 1, 2 and 5) of the transmembrane domain in the MUC1-TRIM46-KRTCAP2 fusion proteins does not change the intracellular localization of these isoforms as all five isoforms are localized in the cytoplasm. Taken together, these fusion isoforms appear to have very different molecular properties as compared to the parental MUC1. MUC1 normally is a transmembrane protein that is heavily glycosylated. However, in breast and ovarian tumors, MUC1 was observed to be underglycosylated and assumed an additional intracellular localization that is associated with poor prognosis [13,16]. In this respect, MUC1-TRIM46-KRTCAP2 fusion isoforms are similar to tumor-associated MUC1 as they are not glycosylated and are localized intracellularly.

One important consequence of the chimeric RNA is that MUC1-TRIM46-KRTCAP2 acquires a new 3’UTR that is lacking the binding site targeted by miR-145, a well-known tumor suppressor that inhibits invasion by silencing MUC1 through binding to its 3’ UTR [19]. This suggests that the MUC1-TRIM46-KRTCAP2 chimeric RNAs escape the regulation normally imposed by miR-145, a factor that could also contribute to tumor progression.

The overexpression of MUC1 in cancers possibly leads to chimeric RNA expression as they both involve the same promoter. However, the considerable variability in the expression of parental MUC1 among normal and tumor samples make it challenging to use it in clinical settings. Since these chimeric RNAs are easily detected in cancer samples and not in non-cancerous ovaries, they could represent a better biomarker as compared to parental MUC1 for detecting HGSC or to monitor treatment progress. Potential detection methods include RT-PCR or antibody against the novel epitope in the fusion proteins. If the fusion proteins are proven to be oncogenic, then they could also serve as a therapeutic target for small molecule drugs.

## 4. Experimental Section

### 4.1. Human High-Grade Serous Ovarian Cancer Samples

Fifty-nine anonymous ovarian cancer tissue samples were obtained from the Tissue Acquisition and Distribution Core of the Dan L. Duncan Cancer Center, Department of Pathology & Immunology, and the Gynecologic Oncology Group under an approved Baylor College of Medicine Institutional Review Board protocol [18]. All tumor samples were confirmed to have greater than 80% serous adenocarcinoma prior to processing. We also obtained 24 non-cancerous ovaries. RNA was extracted from cancer samples and non-cancerous samples using a Ribopure kit (Ambion, Austin, TX, USA) and processed for RT-PCR.

### 4.2. Bioinformatic Identification of Gene Fusions

The analyses were carried out using the high-throughput transcriptome sequencing data of 130 HGSC samples from the TCGA. RNA-Seq reads were processed as described in reference [18]. Briefly, this procedure involved the following filters in order: (1) trimming by base quality score in 5’ to 3’ direction, using 15 as the minimum threshold; and (2) removing reads smaller than 45 basepairs. Reads were first mapped to the transcriptome using Pash 3.0 [20]. Reads pairs mapping to non-overlapping genes with 0 mismatches were preserved as inconsistent reads; reads mapping to the same gene or overlapping genes were discarded from analysis. Reads with at most one end mapping to a gene were further selected, and mapped to the genome using bwa [21]. Again reads mapping to the same gene or overlapping genes were discarded, whereas reads mapping to two different genes were selected. Inconsistent read pairs derived from either transcriptome or genomic mapping were then combined, and non-overlapping gene pairs with at least two read pairs spanning them were selected as candidate gene fusions.

### 4.3. RT-PCR

One microgram of total RNA from either the cancerous or non-cancerous sample was used for each reverse transcription reaction following the protocol described in reference [18]. Briefly, RNA was polyA primed by being incubated with Oligo dT and dNTPs, and denatured at 65 °C. This was followed by the addition of a master-mix of 20 µL total volume containing: 1× superscript buffer, 10 mM DTT, 5 mM Magnesium chloride, RNaseOUT and Superscript III reverse transcriptase. Reactions were then incubated at 50 °C for 50 min. Reactions were terminated by incubation at 85 °C for 5 min. cDNA was then treated with RNase-H for 20 min at 37 °C. Two microliters of cDNA from the above reaction was used as template for PCR using the primers listed in Table S4. PCR master mixes included 3% DMSO and PCR was performed using the follow temperature:

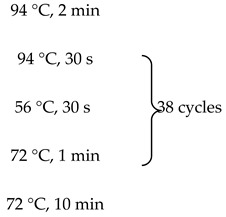


As a negative control, the PCR was performed without any input cDNA.

Densitometric analysis was performed using the digital images. After background correction, the total pixel density was calculated for each band in the gel. Then, a cut-off value significantly higher than the background value was used to determine those samples as positive and negative samples for a particular isoform.

### 4.4. Long Range PCR

To identify the potential genomic breakpoints, we performed long-range PCR on 200 ng of genomic DNA using LA PCR kit (Takara) with the primers listed in Table S4. Reactions were performed according to the manufacturer’s protocols. Two-step PCR was performed with denaturation at 98 °C for 30 s followed by annealing and extension, both at 68 °C for 20 min. The products were run on gels.

### 4.5. Cloning and Transfection

Plasmid constructs were made to express the different MUC1-TRIM46-KRTCAP2 isoforms driven by CMV promoter. Using the primers listed in Table S4, RT-PCR was performed on patient RNA to generate the full-length cDNA products. A C-terminal FLAG tag was added to all PCR products, and they were cloned into the vector HDM [22], which has a CMV promoter. OVCAR8 cell line was transfected with the indicated plasmids or parental HDM vector using the X-tremegene transfection reagent (Roche, Basel, Switzerland) following the manufacturer’s protocol.

### 4.6. Protein Extraction of Transfected Cells and Western Blotting

Two days after the transfection of expression plasmids, proteins were extracted using NETN buffer (NETN composition: 100 mM NaCl, 1 mM EDTA, 20 mM Tris-HCl pH 7.5 and 0.5% NP-40) at either 24 or 48 h. Briefly, cells were washed with PBS, NETN buffer (supplemented with SIGMA protease inhibitor cocktail) was added to the cells, and lysis was allowed to proceed for 5 min on ice. Cells were scraped and collected in eppendorf tubes and centrifuged at 8000 × g for 10 min at 4 °C. The supernatant was collected and used in Western blot analysis.

Western blot analysis was carried out as described in reference [18]. Briefly, equal amounts of lysates were run on a 4%–20% Tris-glycine gel (Bio-rad). Proteins were transferred onto a nitrocellulose membrane using CAPS buffer (VWR) at 100 V for 1 h. The membrane was rinsed with 1× TBS and then blocked with 1× TBST containing 5% nonfat dry milk for 2 h. This was followed by three washes with 1× TBST. The primary antibody (Anti-FLAG; SIGMA F1804) in blocking buffer (5% milk) was incubated with the membrane overnight at 4 °C. After three washes with 1× TBS/T, the membrane was incubated with a secondary antibody (Anti-mouse IgG, Cell Signaling Technology) in blocking buffer for 2 h. This was followed by additional three washes. The membrane was then incubated with detection reagents (Supersignal West Femto from Thermo Scientific, Rockford, IL, USA) and exposed to film.

### 4.7. Deconvolution Fluorescence Microscopy and Immunofluorescence of Transfected Cells

OVCAR8 cells were grown on coverslips and then transfected with MUC1-TRIM46-KRTCAP2 isoforms that were FLAG tagged. Cells were washed with PBS briefly and then fixed in paraformaldehyde for 10 min twice followed by another wash with PBS. Coverslips were then left in triton extraction buffer for 20 min on ice. This was followed by two washes with PBS/0.1% NP-40 for 5 min each time. Coverslips were then incubated with blocking buffer (10% horse serum/PBS/0.1% NP-40) for one hour. Anti-FLAG antibody (SIGMA F1804) was diluted 1:10,000 in buffer (5% horse serum/PBS/0.1% NP-40) and added to coverslips for one hour. This was followed by three washes with PBS/0.1% NP-40. Alexa Fluor 488 Goat Anti-Mouse IgG (Invitrogen A-11001) was diluted 1:200 in buffer (3% horse serum/PBS/0.1% NP-40) and added to coverslips for one hour. This was followed by three washes and staining with DAPI for 10 min. Coverslips were then mounted using Vectashield. Images were taken on a DeltaVision inverted deconvolution/image restoration microscope at the BCM integrated microscopy core facility.

## Supplementary Files

Supplementary File 1
