# Supplementary material for: Aberrant MUC1-TRIM46-KRTCAP2 Chimeric RNAs in High-Grade Serous Ovarian Carcinoma
Source: Cancers (Basel). 2015 Oct 19;7(4):2083–93. doi: 10.3390/cancers7040878 (PMC4695878; doi:10.3390/cancers7040878)
Supplement: Supplementary File 1 [file cancers-07-00878-s001.pdf]

Supplementary Materials

Table S1. RNA junction sequences of identified chimeric RNAs.

Chimera	RNA Fusion Junction Sequence
MUC1-TRIM46	See Table S5
SH2D4A-CSGALNACT1	TTTCCTCATCGATGCCTCTGCAGACGCCTACAGCTTCCTGGGCGTGGACC AGCTACAGCATGCCACCTTGGCGGATTTGGTGGAATATCACAAG*GTGAG CCTCTATCTCGTGCCTGAACACCAGCATGCCCAGCTGGCCGTGG
CCDC19-VSIG8	CAGCAGAAGGAAGTGCAGAACCGGATTGCCACCTTTGAGGAGGGCCGG CGCCTCAAAGAGGAGGCCCAGAAACGCCGTGAGCGCATCGATGAGATC AAGAGGAAAAAGCTTGAAGAGCTGAG*CACTGCTGTCTGCTGTGCGGAT CAACGGGGATGGACAGGAGGTCCTGTACCTGGCAGAAGGTGATAATGTG AGGCTGGGCTGCCCCTACGTCCTGGACCCTGAGGACTATGGTCCCAATG GGCTGGACATCGAGTGGATGCAGGTCAACT
SFPQ-AL831889	TGGTGGTGGCATAGGTTATGAAGCTAATCCTGGCGTTCCACCAGCAACC ATGAGTGGTTCCATGATGGGAAGTGACATG*GTAAGAATGATTGATGTTG GCTGATATTGGAGTGCTCATTCACATGAAGTGGATAGATACTTCTCAAGA CATCACACAGCGTGAGTCAATCAAGGAGGGAAGCCACAAGCAGACTGA CAACGTTTCTAGGATCAGGTGAGCTGTGTCCAGAAA
CS-CNPY2	CTGCTCACCTTGGTTCCCAAGAGCCGGGCGGCCGCA*CTCCGACTCAGAT TTTGAACGGATGTTTGCATGTATACCACAGTGCTACATTAAAAACCAGTC ATGTGGATGTGGAGAAGGGTGCTGATCAGGACTCCCTGTTTGCTAACAA ATTCTACAGCAGAAACAAAGTATTAAGGTTTGCAGCCAGGAAGCAAGTG CATGGGGAA
SLC16A3-CSNK1D	TTCAAGGAGCTCATACAGGAGTTTGGGATCGGCTACAGCGACACAGCCT GGATCTCCTCCATCCTGCTGGCCATGCTCTACGGGACAG*GGGTCCTGCT GTCACCCAGGCTGGAAGGCAGTGGCACCATCACAGCTCCCTGCAGCCTT AACCTCCCGAACTCTAGCCATCCTCCTGCCTCAGCCTCCAGAGTAGCTGA GACTACAGATGTAAGC
SPINT2-YIF1B	CCCACGCTGGTACTTTGACGTGGAGAGGAACTCCTGCAATAACTTCATCT ATGGAGGCTGCCGGGGCAATAAGAACAGCTACCGCTCTGAGGAGGCCT GCATGCTCCGCTGCTTCCGCCAGCAGGAGAATCCTCCCCTGCCCCTTGGC TCAAAGG*CCCTGGAGCCCACCTCCCAGAAGCCCGGTGTGGG
CTSC-RAB38	TTGCCAACATCTTGGGACTGGAGAAATGTTCATGGTATCAATTTTGTCAG TCCTGTTCGAAACCAAG*GTCAAGAAAGATTTGGAAACATGACGAGGGT CTATTACCGAGAAGCTATGGGTGCATTTATTGTCTTCGATGTCACCAGGC CAGCCACATTTGAAGCAGTGGCAAAGTGGAAAA
JAK3-INSL3	CCGCCTCTTGGAACTGCTGGAGGAGGGCCAGAGGCTGCCGGCGCCTCCT GCCTGCCCTGCTGAG*GTGAGTTGCTACAGTGGCTGGAGAGACGACATCT GCTCCATGGGCTGGTGGCCGACAGTAATCTCACGCT

The nine validated chimeric RNAs along with their RNA fusion junctions are shown. The RNA fusion junction is represented by "*".

Table S2. Recurrence profile of identified chimeric RNAs. The validated chimeric RNAs are shown along with their recurrence in the 130 TCGA sample cohort by bioinformatics analysis. Since TCGA transcriptome sequencing collection does not contain organ specific controls, we examined these nine chimeric RNAs using our in-house non-cancerous ovary samples by RT-PCR to answer the question of whether these candidates are also present in non-cancerous tissue.

Chimera	Type	Chromosome	Recurrence among 130 TCGA Samples	Recurrence among 11 Non-Cancer Donor Ovary Samples
MUC1-TRIM46	Intrachromosomal	1	9	0
SH2D4A-CSGALNACT1	Intrachromosomal	8	22	11
CCDC19-VSIG8	Intrachromosomal	1	10	1
SFPQ-AL831889	Intrachromosomal	1	98	11
CS-CNPY2	Intrachromosomal	12	23	n.d.
SLC16A3-CSNK1D	Intrachromosomal	17	18	5
SPINT2-YIF1B	Intrachromosomal	19	55	11
CTSC-RAB38	Intrachromosomal	11	6	11
JAK3-INSL3	Intrachromosomal	19	7	11

n.d.: not determined.

Table S3. Paired chimeric reads for *MUC1-TRIM46* identified from TCGA database. These 25 reads were obtained from nine patient samples. These nine patient sample IDs are: TCGA-04-1348, TCGA-10-0931, TCGA-13-0720, TCGA-13-1403, TCGA-13-1482, TCGA-23-1122, TCGA-24-0979, TCGA-24-1560 and TCGA-24-1604.

Read 1 (Aligning to MUC1 Gene)	Read 2 (Aligning to TRIM46 Gene)
CATTTCAAACCTCCAGTTTAATTCCTCTCTGGAAGATCCCAGCACCGACTACTACCAAGAGCTGCAGAGAGACAT	TGCGACAGGGAGGAAATAATGCGTCGAGGGGGCATCCTCGGCCACCGCCCTGGTCCTCAGGCTTTCCTCGTACCT
CGAGTACCCCACCTACCACACCCATGGGCGCTATGTGCCCCCTAGCAGTACCGATCGTAGCCCCTATGAGAAGAG	GAGGAAATAATGCGTCGAGGGGGCATCCTCGGCCACCGCCCTGGTCCTCAGGCTTTCCTCGTACCTGGTGCGTCT
GATACCTACCATCCTATGAGCGAGTACCCCACCTACCACACCCATGGGCGCTATGTGCCCCCTAGCAGTACCGAT	GCAGCGCCCTCTTGCGACAGGGAGGAAATAATGCGTCGAGGGGGCATCCTCGGCCACCGCCCTGGTCCTCAGGCT
ATCCTATGAGCGAGTACCCCACCTACCACACCCATGGGCGCTATGTGCCCCCTAGCAGTACCGATCGTAGCCCCT	TCGAGGGGGCATCCTCGGCCACCGCCCTGGTCCTCAGGCTTTCCTCGTACCTGGTGCGTCTGCTGCCACTCTTCT
GAAGAATGCTTTTAATTCCTCTCTGGAAGATCCCAGCACCGACTACTACCAAGAGCTGCAGAGAGACATTTCTGA	AGGGGGCATCCTCGGCCACCGCCCTGGTCCTCAGGCTTTCCTCGTACCTGGTGCGTCTGCTGCCACTCATTTCAG
ATCCTATGAGCGAGTACCCCACCTACCACACCCATGGGCGCTATGTGCCCCCTAGCAGTACCGATCGTAGCCCCT	CCTGGACGCAGCGCCCTCTTGCGACAGGGAGGAAATAATGCGTCGAGGGGGCATCCTCGGCCACCGCCCTGGTCC
CGGGATACCTACCATCCTATGAGCGAGTACCCCACCTACCACACCCATGGGCGCTATGTGCCCCCTAGCAGTACC	GTCGAGGGGGCATCCTCGGCCACCGCCCTGGTCCTCAGGCTTTCCTCGTACCTGGTGCGTCTGCTGCCACTCTTC
GAATTAAACTGGAGGTTTGAAATGTGAAAAGACAGGAAAAAGAAAGAGACCCCAGTAGACAACTGGGGAGAAGTG	GGTACGAGGAAAGCCTGAGGACCAGGGCGGTGGCCGAGGATGCCCCCTCGACGCATTATTTCCTCCCTGTCGCAA
GAGTACCCCACCTACCACACCCATGGGCGCTATGTGCCCCCTAGCAGTACCGATCGTAGCCCCTATGAGAAGAGT	TCTCCTCTTTCTTCCTCTCAGTGCCTGGACGCAGCGCCCTCTTGCGACAGGGAGGAAATAATGCGTCGAGGGGGC

Table S3. *Cont.*

Read 1 (Aligning to MUC1 Gene)	Read 2 (Aligning to TRIM46 Gene)
GTCAGTGCCGCCGAAAGAACTACGGCAGCT GGACATCTTTCCAGCCCGGGATACCTACCATC CTATGAGCGAG	GGACGCAGCGCCCTCTTGCGACAGGGAGGA AATAATGCGTCGAGGGGGCATCCTCGGCCAC CGCCCTGGTCCTCA
AGCTGGACATCTTTCCAGCCCGGGATACCTAC CATCCTATGAGCGAGTACCCCACCTACCACAC CCATGGGCGCT	GGAAATAATGCGTCGAGGGGGCATCCTCGG CCACCGCCCTGGTCCTCAGGCTTTCCTCGTAC CTGGTGCGTCTGC
CCACCTGGGGACAGGATGTCACCTCGGTCCCA GTCACCAGGCCAGCCCTGGGCTCCACCACCC	GCGCTCAGCGCTGCTTCTCCCTCTCTTTGTTTG TAGGCTGTGGCCACCGCGGACTCTGCTCCGG AGCACCCCAGG
GTACCCCACCTACCACACCCATGGGCGCTATG TGCCCCCTAGCAGTACCGATCGTAGCCCCTAT GAGAAGAGTGG	CTCTTGCGACAGGGAGGAAATAATGCGTCGA GGGGGCATCCTCGGCCACCGCCCTGGTCCTC AGGCTTTCCTCGT
GGCTACGATCGGTACTGCTAGGGGGCACATAG CGCCCATGGGTGTGGTAGGTGGGGTACTCGCT CATAGGATGG	TGCGTCGAGGGGGCATCCTCGGCCACCGCCC TGGTCCTCAGGCTTTCCTCGTACCTGGTGCGT CTGCTGCCACTC
TGTCTTTTCACATTTCAAACCTCCAGTTTAATTC CTCTCTGGAAGATCCCAGCACCGACTACTACC AAGAGCTGC	GAAATAATGCGTCGAGGGGGCATCCTCGGCC ACCGCCCTGGTCCTCAGGCTTTCCTCGTACCT GGTGCGTCTGCT
ACCACACCCATGGGCGCTATGTGCCCCCTAGC AGTACCGATCGTAGCCCCTATGAGAAGAGTGG CAG	AGGGAGGAAATAATGCGTCGAGGGGGCATC CTCGGCCACCGCCCTGGTCCTCAGGCTTTCCT CGTACCTGGTGCG
CCTACCACACCCATGGGCGCTATGTGCCCCCT AGCAGTACCGATCGTAGCCCCTATGAGAAGAG TGGCAG	GCCCTCTTGCGACAGGGAGGAAATAATGCGT CGAGGGGGCATCCTCGGCCACCGCCCTGGTC CTCAGGCTTTCCT
CCATCCTATGAGCGAGTACCCCACCTACCACA CCCATGGGCGCTATGTGCCCCCTAGCAGTACC GATCGTAGCCC	CAGTGCCTGGACGCAGCGCCCTCTTGCGACA GGGAGGAAATAATGCGTCGAGGGGGCATCC TCGGCCACCGCCCT
ATGAGCGAGTACCCCACCTACCACACCCATGG GCGCTATGTGCCCCCTAGCAGTACCGATCGTA GCCCCTATGAG	GTCGAGGGGGCATCCTCGGCCACCGCCCTGG TCCTCAGGCTTTCCTCGTACCTGGTGCGTCTG CTGCCACTCTTC
ACAATGGCCAGCGCAACCAGAACACAGACCA GCACCAGCAGCGCGATG	ATGCGTCGAGGGGGCATCCTCGGCCACCGCC CTGGTCCTCAGGCTTTCCTCGTACCTGGTGCG TCTGCTGCCACT
GTCTTTTCACATTTCAAACCTCCAGTTTAATTCC TCTCTGGAAGATCCCAGCACCGACTACTACCA AGAGCTGCA	AATAATGCGTCGAGGGGGCATCCTCGGCCAC CGCCCTGGTCCTCAGGCTTTCCTCGTACCTGG TGCGTCTGCTGC
ACCATCCTATGAGCGAGTACCCCACCTACCAC ACCCATGGGCGCTATGTGCCCCCTAGCAGTAC CGATCGTAGCC	GGAGGAAATAATGCGTCGAGGGGGCATCCT CGGCCACCGCCCTGGTCCTCAGGCTTTCCTC
CTACCCAGAGAAGTTCAGTGCCCAGCTCTACT GAGAAGAATGCTTTTAATTCCTCTCTGGAAGAT CCCAGCACCG	ATAATGCGTCGAGGGGGCATCCTCGGCCACC GCCCTGGTCCTCAGGCTTTCCTCGTACCTGGT GCGTCTGCTGCC
GACATCTTTCCAGCCCGGGATACCTACCATCCT ATGAGCGAGTACCCCACCTACCACACCCATGG GCGCTATGTG	GGAAATAATGCGTCGAGGGGGCATCCTCGG CCACCGCCCTGGTCCTCAGGCTTTCCTCGTAC CTGGTGCGTCTGC
CTCCAGTTTAATTCCTCTCTGGAAGATCCCAGC ACCGACTACTACCAAGAGCTGCAGAGAGACA TTTCTGAAATG	GCCTGGACGCAGCGCCCTCTTGCGACAGGGA GGAAATAATGCGTCGAGGGGGCATCCTCGG CCACCGCCCTGGTC

Table S4. Primers used in this study.

Primers	Sequence 5'-> 3'
TRIM46-MUC1 F1	AAGATCCCAGCACCGACTACT
TRIM46-MUC1 R1	AGGGAGGAAATAATGCGTCGA
MUC1 fwd	GCCCGCTCCACCTCTCAAGCAGCCAGCGCCTGCCTGAATCTGTTCT
KRTCAP2 R9	GTCAGTTTCTCTTCTTGCTCT
E-S-N muc1 fwd new	GGCAAAGAATTCATAATTAACCGCGGGCGGCCGCCCGCTCCACCTC<br>TCAAGCAGCCAGCGCCTGCCTGAATCTGTTCT
Bam-Stop-FLAG Muc1 rev	TTGGATCCCGTCATTACTTGTCGTCATCGTCTTT<br>GTAGTCTTGAAGGCAGTGAGCGAGAACA

Table S5. MUC1-TRIM46-KRTCAP2 isoforms and RNA fusion junctions. MUC1 sequence is in red, TRIM46 in blue and KRTCAP2 sequence is in green. Lowercase letters represent 5′ and 3′ UTR sequences. Start and stop codons are shown in Bold. "*" Indicates fusion junction between MUC1 and TRIM46 while "^" indicates fusion junction between TRIM46 and KRTCAP2.

MUC1-TRIM46-KRTCAP2 isoform 1 complete cDNA:	acgctccacctctcaagcagccagcgcctgcctgaatctgttctgcccctccccacccatttcaccaccaccATGACACCGG<br>GCACCCAGTCTCCTTTCTTCCTGCTGCTGCTCCTCACAGTGCTTACAGCTACCACAG<br>CCCCTAAACCCGCAACAGTTGTTACGGGTTCTGGTCATGCAAGCTCTACCCCAGGT<br>GGAGAAAAGGAGACTTCGGCTACCCAGAGAAGTTCAGTGCCCAGCTCTACTGAGA<br>AGAATGCTTTGTCTACTGGGGTCTCTTTCTTTTTCCTGTCTTTTCACATTTCAAACCTC<br>CAGTTTAATTCCTCTCTGGAAGATCCCAGCACCGACTACTACCAAGAGCTGCAGAG<br>AGACATTTCTGAAATG*AGTGGC<br>AGCAGACGCACCAGGTACGAGGAAAGCCTGAGGACCAGGGCGGTGGCCGAGGAT<br>GCCCCCTCGACGCATTATTTCCTCCCTGTCGCAAGAGGGCGCTGCGTCCAGGCACT<br>GAGAGGAAGAAAGAGGAGAACGCGAGGAGTAGCAAGTCCGCG^TGGTGGGTACG<br>GGCACCTCGCTGGCGCTCTCCTCCCTCCTGTCCCTGCTGCTCTTTGCTGGGATGCAG<br>ATGTACAGCCGTCAGCTGGCCTCCACCGAGTGGCTCACCATCCAGGGCGGCCTGCT<br>TGGTTCGGGTCTCTTCGTGTTCTCGCTCACTGCCTTCAATAAtctggagaatcttgtctttggcaaa<br>ggattccaagcaaagatcttccctgagattctcctgtgcctcctgttggctctctttgcatctggcctcatccaccgagtctgtgtcacca<br>cctgcttcatcttctccatggttggtctgtactacatcaacaagatctcctccaccctgtaccaggcagcagctccagtcctcacaccagc<br>caaggtcacaggcaagagcaagaagagaaactgaccctgaatgttcaataaagttgattctttgt
MUC1-TRIM46-KRTCAP2 isoform 2 complete cDNA:	cgctccacctctcaagcagccagcgcctgcctgaatctgttctgcccctccccacccatttcaccaccacc**ATG**ACACCGGG<br>CACCCAGTCTCCTTTCTTCCTGCTGCTGCTCCTCACAGTGCTTACAGCTACCACAGC<br>CCCTAAACCCGCAACAGTTGTTACGGGTTCTGGTCATGCAAGCTCTACCCCAGGTG<br>GAGAAAAGGAGACTTCGGCTACCCAGAGAAGTTCAGTGCCCAGCTCTACTGAGAA<br>GAATGCTTTTAATTCCTCTCTGGAAGATCCCAGCACCGACTACTACCAAGAGCTGC<br>AGAGAGACATTTCTGAAATGGCTGTCTGTCAGTGCCGCCGAAAGAACTACGGGCA<br>GCTGGACATCTTTCCAGCCCGGGATACCTACCATCCTATGAGCGAGTACCCCACCT<br>ACCACACCCATGGGCGCTATGTGCCCCCTAGCAGTACCGATCGTAGCCCCTATGAG<br>AAG*AGTGGCAGCAGACGCACCAGGTACGAGGAAAGCCTGAGGACCAGGGCGGT<br>GGCCGAGGATGCCCCCTCGACGCATTATTTCCTCCCTGTCGCAAGAGGGCGCTGCG<br>TCCAGGCACTGAGAGGAAGAAAGAGGAGAACGCGAGGAGTAGCAAGTCCGCG^T<br>GGTGGGTACGGGCACCTCGCTGGCGCTCTCCTCCCTCCTGTCCCTGCTGCTCTTTGC<br>TGGGATGCAGATGTACAGCCGTCAGCTGGCCTCCACCGAGTGGCTCACCATCCAG<br>GGCGGCCTGCTTGGTTCGGGTCTCTTCGTGTTCTCGCTCACTGCCTTCAA**TAA**tctggag<br>aatcttgtctttggcaaaggattccaagcaaagatcttccctgagattctcctgtgcctcctgttggctctctttgcatctggcctcatcca<br>ccgagtctgtgtcaccacctgcttcatcttctccatggttggtctgtactacatcaacaagatctcctccaccctgtaccaggcagcagct<br>ccagtcctcacaccagccaaggtcacaggcaagagcaagaagagaaactgaccctgaatgttcaataaagttgattctttgt

Table S5. *Cont*.

MUC1-TRIM46-KRTCAP2 isoform 3 complete cDNA:	cgctccacctctcaagcagccagcgcctgcctgaatctgttctgcccctccccacccatttcaccaccacc**ATG**ACACCGGG<br>CACCCAGTCTCCTTTCTTCCTGCTGCTGCTCCTCACAGTGCTTACAGCTACCACAGC<br>CCCTAAACCCGCAACAGTTGTTACGGGTTCTGGTCATGCAAGCTCTACCCCAGGTG<br>GAGAAAAGGAGACTTCGGCTACCCAGAGAAGTTCAGTGCCCAGCTCTACTGAGAA<br>GAATGCTTTTAATTCCTCTCTGGAAGATCCCAGCACCGACTACTACCAAGAGCTGC<br>AGAGAGACATTTCTGAAATGTCTGGGGCTGGGGTGCCAGGCTGGGGCATCGCGCT<br>GCTGGTGCTGGTCTGTGTTCTGGTTGCGCTGGCCATTGTCTATCTCATTGCCTTGGCT<br>GTCTGTCAGTGCCGCCGAAAGAACTACGGGCAGCTGGACATCTTTCCAGCCCGGG<br>ATACCTACCATCCTATGAGCGAGTACCCCACCTACCACACCCATGGGCGCTATGTG<br>CCCCCTAGCAGTACCGATCGTAGCCCCTATGAGAAG*AGTGGCAGCAGACGCACC<br>AGGTACGAGGAAAGCCTGAGGACCAGGGCGGTGGCCGAGGATGCCCCCTCGACG<br>CATTATTTCCTCCCTGTCGCAAGAGGGCGCTGCGTCCAGGCACTGAGAGGAAGAA<br>AGAGGAGAACGCGAGGAGTAGCAAGTCCGCG^TGGTGGGTACGGGCACCTCGCTG<br>GCGCTCTCCTCCCTCCTGTCCCTGCTGCTCTTTGCTGGGATGCAGATGTACAGCCGT<br>CAGCTGGCCTCCACCGAGTGGCTCACCATCCAGGGCGGCCTGCTTGGTTCGGGTCT<br>CTTCGTGTTCTCGCTCACTGCCTTCAA**TAA**tctggagaatcttgtctttggcaaaggattccaagcaaagatc<br>ttccctgagattctcctgtgcctcctgttggctctctttgcatctggcctcatccaccgagtctgtgtcaccacctgcttcatcttctccatgg<br>ttggtctgtactacatcaacaagatctcctccaccctgtaccaggcagcagctccagtcctcacaccagccaaggtcacaggcaaga<br>gcaagaagagaaactgaccctgaatgttcaataaagttgattctttgt
MUC1-TRIM46-KRTCAP2 isoform 4 complete cDNA:	Gcgcctgcctgaatctgttctgcccctccccacccatttcaccaccaccatgacaccgggcacccagtctcctttcttcctgctgctgct<br>cctcacagtgcttacaggtgaggggcacgaggtggggagtgggctgccctgcttaggtggtcttcgtggtctttctgtgggttttgct<br>ccctggcagatggcaccatgaagttaagctaccacagcccctaaacccgcaacagttgttacgggttctggtcatgcaagctctaccc<br>caggtggagaaaaggagacttcggctacccagagaagttcagtgcccagctctactgagaagaatgcttttaattcctctctggaag<br>atcccagcaccgactactaccaagagctgcagagagacatttctgaa**ATGTGA**gtgatgtgccatttcctttctctgcccagtct<br>ggggctggggtgccaggctggggcatcgcgctgctggtgctggtctgtgttctggttgcgctggccattgtctatctcattgccttgg<br>ctgtctgtcagtgccgccgaaagaactacgggcagctggacatctttccagcccgggatacctaccatcctatgagcgagtacccca<br>cctaccacacccatgggcgctatgtgccccctagcagtaccgatcgtagcccctatgagaag*agtggcagcagacgcaccaggta<br>cgaggaaagcctgaggaccagggcggtggccgaggatgccccctcgacgcattatttcctccctgtcgcaagagggcgctgcgtc<br>caggcactgagaggaagaaagaggagaacgcgaggagtagcaagtccgcg^tggtgggtacgggcacctcgctggcgctctc<br>ctccctcctgtccctgctgctctttgctgggatgcagatgtacagccgtcagctggcctccaccgagtggctcaccatccagggcggc<br>ctgcttggttcgggtctcttcgtgttctcgctcactgccttcaataatctggagaatcttgtctttggcaaaggattccaagcaaagatctt<br>ccctgagattctcctgtgcctcctgttggctctctttgcatctggcctcatccaccgagtctgtgtcaccacctgcttcatcttctccatggtt<br>ggtctgtactacatcaacaagatctcctccaccctgtaccaggcagcagctccagtcctcacaccagccaaggtcacaggcaagagc<br>aagaagagaaactgaccctgaatgttcaataaagttgattctttgt
MUC1-TRIM46-KRTCAP2 isoform 5 complete cDNA:	ataattaaccgcgggcggccgccgctccacctctcaagcagccagcgcctgcctgaatctgttctgcccctccccacccatttcacca<br>ccacc**ATG**ACACCGGGCACCCAGTCTCCTTTCTTCCTGCTGCTGCTCCTCACAGTGCTT<br>ACAGTTGTTACGGGTTCTGGTCATGCAAGCTCTACCCCAGGTGGAGAAAAGGAGA<br>CTTCGGCTACCCAGAGAAGTTCAGTGCCCAGCTCTACTGAGAAGAATGCTGCTGTC<br>TGTCAGTGCCGCCGAAAGAACTACGGGCAGCTGGACATCTTTCCAGCCCGGGATA<br>CCTACCATCCTATGAGCGAGTACCCCACCTACCACACCCATGGGCGCTATGTGCCC<br>CCTAGCAGTACCGATCGTAGCCCCTATGAGAAG*AGTGGCAGCAGACGCACCAGG<br>TACGAGGAAAGCCTGAGGACCAGGGCGGTGGCCGAGGATGCCCCCTCGACGCATT<br>ATTTCCTCCCTGTCGCAAGAGGGCGCTGCGTCCAGGCACTGAGAGGAAGAAAGAG<br>GAGAACGCGAGGAGTAGCAAGTCCGCG^TGGTGGGTACGGGCACCTCGCTGGCGC<br>TCTCCTCCCTCCTGTCCCTGCTGCTCTTTGCTGGGATGCAGATGTACAGCCGTCAGC<br>TGGCCTCCACCGAGTGGCTCACCATCCAGGGCGGCCTGCTTGGTTCGGGTCTCTTCG<br>TGTTCTCGCTCACTGCCTTCAA**TAA**tctggagaatcttgtctttggcaaaggattccaagcaaagatcttccctga<br>gattctcctgtgcctcctgttggctctctttgcatctggcctcatccaccgagtctgtgtcaccacctgcttcatcttctccatggttggtctg<br>tactacatcaacaagatctcctccaccctgtaccaggcagcagctccagtcctcacaccagccaaggtcacaggcaagagcaagaa<br>gagaaactgaccctgaatgttcaataaagttgattctttgt

Table S5. *Cont*.

MUC1-TRIM46-KRTCAP2 isoform 6 complete cDNA:	Cgctccacctctcaagcagccagcgcctgcctgaatctgttctgcccctccccacccatttcaccaccaccatgacaccgggcaccc<br>agtctcctttcttcctgctgctgctcctcacagtgcttacagctaccacagcccctaaacccgcaacagttgttacgggttctggtcatgc<br>aagctctaccccaggtggagaaaaggagacttcggctacccagagaagttcagtgcccagctctactgagaagaatgctatcccag<br>caccgactactaccaagagctgcagagagacatttctgaa**ATG**TTTTTGCAGATTTATAAACAAGGGGGT<br>TTTCTGGGCCTCTCCAATATTAAGTTCAGGCCAGGATCTGTGGTGGTACAATTGACT<br>CTGGCCTTCCGAGAAGGTACCATCAATGTCCACGACGTGGAGACACAGTTCAATC<br>AGTATAAAACGGAAGCAGCCTCTCGATATAACCTGACGATCTCAGACGTCAGCGT<br>GAGTGATGTGCCATTTCCTTTCTCTGCCCAGTCTGGGGCTGGGGTGCCAGGCTGGG<br>GCATCGCGCTGCTGGTGCTGGTCTGTGTTCTGGTTGCGCTGGCCATTGTCTATCTCAT<br>TGCCTTGGCTGTCTGTCAGTGCCGCCGAAAGAACTACGGGCAGCTGGACATCTTTC<br>CAGCCCGGGATACCTACCATCCTATGAGCGAGTACCCCACCTACCACACCCATGG<br>GCGCTATGTGCCCCCTAGCAGTACCGATCGTAGCCCCTATGAGAAG*AGTGGCAGC<br>AGACGCACCAGGTACGAGGAAAGCCTGAGGACCAGGGCGGTGGCCGAGGATGCC<br>CCCTCGACGCATTATTTCCTCCCTGTCGCAAGAGGGCGCTGCGTCCAGGCACTGAG<br>AGGAAGAAAGAGGAGAACGCGAGGAGTAGCAAGTCCGCG^TGGTGGGTACGGGC<br>ACCTCGCTGGCGCTCTCCTCCCTCCTGTCCCTGCTGCTCTTTGCTGGGATGCAGATG<br>TACAGCCGTCAGCTGGCCTCCACCGAGTGGCTCACCATCCAGGGCGGCCTGCTTGG<br>TTCGGGTCTCTTCGTGTTCTCGCTCACTGCCTTCAA**TAA**tctggagaatcttgtctttggcaaaggatt<br>ccaagcaaagatcttccctgagattctcctgtgcctcctgttggctctctttgcatctggcctcatccaccgagtctgtgtcaccacctgct<br>tcatcttctccatggttggtctgtactacatcaacaagatctcctccaccctgtaccaggcagcagctccagtcctcacaccagccaagg<br>tcacaggcaagagcaagaagagaaactgaccctgaatgttcaataaagttgattctttgt

Table S6. Frequency of occurrence of different MUC1-TRIM46-KRTCAP2 isoforms determined using in-house HGSC samples. RT-PCR data from Figure 3A was utilized for densitometric analysis and then the samples positive for each isoform were determined.

MUC1-KRTCAP2 Isoform	Frequency of Occurrence among 59 HGSC Samples
Isoform 1	64%
Isoform 2	47%
Isoform 3	17%
Isoform 4	34%
Isoform 5	34%
Isoform 6	20%

For Isoform 1, the following HGSC samples are considered positive: S4,5,7,11,12,14,15,16,20,21,22,23, 46,49,50,51,52,53,54,57,58,60,61,62,63,64,65,66,67,70,71,72,73,74; For Isoform 2, the following samples are considered positive: S1,2,3,4,5,7,9,11,12,15,23,49,53,54,55,61,62,63,64,65,66,67,71,73,74,75,78,82; For Isoform 3, the following samples are considered positive: S46,49,53,55,61,62,63,71,73,75; For Isoform 4, the following samples are considered positive: S1,3,4,5,7,12,15,20,23,46,49,52,55,57,61,62,72,73,75,82; For Isoform 5, the following samples are considered positive: S1,3,4,5,7,12,15,20,23,46,49,52,55,57,61, 62,72,73,75,82; For Isoform 6, the following samples are considered positive: S1,5,12,46,49,55,61,62,72, 73,75,82.

Table S7. MUC1-TRIM46-KRTCAP2 isoforms protein sequence. MUC1 sequence is in red, TRIM46 in blue and KRTCAP2 sequence is in green. “*” Indicates fusion junction between MUC1 and TRIM46 while "^" indicates fusion junction between TRIM46 and KRTCAP2.

Isoform 1: 210 aa	MTPGTQSPFFLLLLLTVLTATTAPKPATVVTGSGHASSTPGGEKETSATQRSSVPSSTEKNALSTGVSFFFLSFHISNLQFNSSLEDPSTDYYQELQRDISEM*SGSRRTRYEESLRTRAVAEDAPSTHYFLPVARGRCVQALRGRKRRTRGVASPR^GGYGHLAGALLPPVPAALCWDADVQPSAGLHRVAHHPGRPAWFGSLRVLAHCLQ
Isoform 2: 242 aa	MTPGTQSPFFLLLLLTVLTATTAPKPATVVTGSGHASSTPGGEKETSATQRSSVPSSTEKNAFNSSLEDPSTDYYQELQRDISEMAVCQCRRKNYGQLDIFPARDTYHPMSEYPTYHTHGRYVPPSSTDRSPYEK*SGSRRTRYEESLRTRAVAEDAPSTHYFLPVARGRCVQALRGRKRRTRGVASPR^GGYGHLAGALLPPVPAALCWDADVQPSAGLHRVAHHPGRPAWFGSLRVLAHCLQ
Isoform 3: 272 aa	MTPGTQSPFFLLLLLTVLTATTAPKPATVVTGSGHASSTPGGEKETSATQRSSVPSSTEKNAFNSSLEDPSTDYYQELQRDISEMSGAGVPGWGIALLVLVCVLVALAIVYLIALAVCQCRRKNYGQLDIFPARDTYHPMSEYPTYHTHGRYVPPSSTDRSPYEK*SGSRRTRYEESLRTRAVAEDAPSTHYFLPVARGRCVQALRGRKRRTRGVASPR^GGYGHLAGALLPPVPAALCWDADVQPSAGLHRVAHHPGRPAWFGSLRVLAHCLQ
Isoform 4: It is predicted to yield no protein since the annotated start codon is immediately followed by a stop codon.	
Isoform 5: 210 aa	MTPGTQSPFFLLLLLTVLTVVTGSGHASSTPGGEKETSATQRSSVPSSTEKNAAVCQCRRKNYGQLDIFPARDTYHPMSEYPTYHTHGRYVPPSSTDRSPYEK*SGSRRTRYEESLRTRAVAEDAPSTHYFLPVARGRCVQALRGRKRRTRGVASPR^GGYGHLAGALLPPVPAALCWDADVQPSAGLHRVAHHPGRPAWFGSLRVLAHCLQ
Isoform 6: 263 aa	MFLQIYKQGGFLGLSNIKFRPGSVVVQLTLAFREGTINVHDVETQFNQYKTEAASRYNLTISDVSVSDVPFPFSAQSGAGVPGWGIALLVLVCVLVALAIVYLIALAVCQCRRKNYGQLDIFPARDTYHPMSEYPTYHTHGRYVPPSSTDRSPYEK*SGSRRTRYEESLRTRAVAEDAPSTHYFLPVARGRCVQALRGRKRRTRGVASPR^GGYGHLAGALLPPVPAALCWDADVQPSAGLHRVAHHPGRPAWFGSLRVLAHCLQ

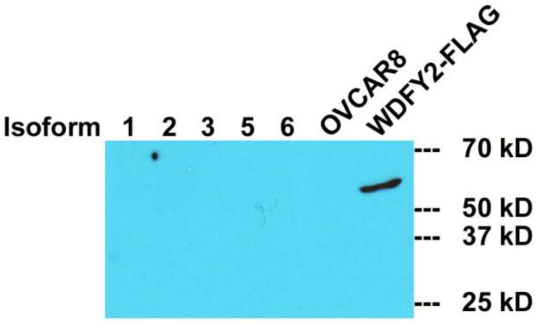

Figure S1. MUC1-TRIM46-KRTCAP2 isoforms are not secreted into the cellular media. MUC1-TRIM46-KRTCAP2 cDNA constructs with FLAG tag were transfected into OVCAR8. 48 hours after transfection, the cellular media was collected and was analyzed by western blotting with a FLAG antibody. None of the isoforms are detectable in the cellular media. WDFY2-FLAG is used as a positive control for the western blot.

© 2015 by the authors; licensee MDPI, Basel, Switzerland. This article is an open access article distributed under the terms and conditions of the Creative Commons by Attribution (CC-BY) license (http://creativecommons.org/licenses/by/4.0/).
